# Sickness absence among 299484 blue-collar workers in the trade industry during the Covid-19 pandemic: Kristin Farrants

**DOI:** 10.1093/eurpub/ckac131.277

**Published:** 2022-10-25

**Authors:** K Farrants, K Alexanderson

**Affiliations:** Division of Insurance Medicine, Department of Clinical Neuroscience, Karolinska Institutet, Stockholm, Sweden

## Abstract

**Background:**

Many blue-collar workers in the trade and retail industries have jobs that make it hard to avoid contacts with other people, which may have increased their risk for sickness absence (SA) during the Covid-19 pandemic. The aim was to investigate rates of SA and sociodemographic and occupational differences in risk of SA during the Covid-19 pandemic.

**Methods:**

A prospective cohort study of all blue-collar workers in Sweden in the trade and retail industry aged 18-67 in 2018 (n = 299 484), followed 5 years (2016-2020) using linked microdata from nationwide registers. Descriptive statistics of rate of workers having had at least one SA-spell >14 days were calculated, and logistic regression was used to calculate odds ratios (OR) and 95% confidence intervals (CI) of having SA due to Covid-19 or related diagnoses (some infectious, respiratory, and symptom-based diagnoses).

**Results:**

Their SA rates increased from fluctuating between 7.6%-8.2% in 2016-2019 to 10.0% in 2020. 0.05% had SA due to Covid-19 and 2.2% had SA due to Covid-19 or related diagnoses. Factors associated with having SA due to Covid-19 or related diagnoses were older age (OR age 55-64: 3.41, CI 3.04-3.82 compared to 18-25) and only elementary education (OR 1.50, CI 1.37-1.64 compared to university/college). Warehouse and terminal staff (reference category) was the occupational group with the highest risk of SA/DP due to Covid-19 or related diagnoses. Cashiers had the second highest risk, with CIs that overlapped 1 (OR 0.91, CI 0.77-1.06). All other occupational groups had significantly lower ORs (0.48-0.78).

**Conclusions:**

The SA rates increased slightly during the Covid-19 pandemic. Within the trade and retail industry, the warehouse and terminal staff was the occupational group with the highest risk of SA due to Covid-19 or related diagnoses.

**Key messages:**

• The rates of SA among blue-collar workers in the trade and retail industry increased slightly during the first year of the Covid-19 pandemic.

• Warehouse and terminal staff and cashiers were the occupational groups with the highest risk of SA due to Covid-19 or related diagnoses among blue-collar workers in the trade and retail industry.

